# Milk Oligosaccharides From Different Cattle Breeds Influence Growth-Related Characteristics of Intestinal Cells

**DOI:** 10.3389/fnut.2019.00031

**Published:** 2019-03-28

**Authors:** Sabine Kuntz, Silvia Rudloff, Clemens Kunz

**Affiliations:** ^1^Institute of Nutritional Sciences, Justus-Liebig-University Giessen, Giessen, Germany; ^2^Department of Pediatrics, Justus-Liebig-University Giessen, Giessen, Germany

**Keywords:** bovine milk oligosaccharides, BMO, interbreed variation, cell cycle dynamics, differentiation, EGFR-ERK signaling

## Abstract

Oligosaccharides are present in human milk (HMO) in large amounts and in a high variety: Among other functions they are considered to influence the gut microbiota and gut maturation in infants. Due to the large volume of milk available bovine milk oligosaccharides (BMO) may be an alternative source of functional ingredients to potentially mimic HMO functions. Thus, we investigated direct effects of bovine milk oligosaccharides (BMO) from different cattle breeds on proliferation, differentiation and apoptosis in transformed (HT-29 and Caco-2) and non-transformed human intestinal cells (HIE cells). We observed a profound growth-inhibition effect induced by all BMO isolates in HT-29, Caco-2, and HIE cells in a dose-dependent manner. The effects varied not only between cell lines, i.e., HT-29 and Caco-2 cells were more sensitive than HIE cells, but also between the cattle breeds. Regarding the induction of differentiation, BMO induced differentiation only in HIE cells without affecting apoptosis. Cell cycle analysis via flow cytometry showed that growth inhibition was associated with a G2/M arrest in all cell lines. Expression levels detected by quantitative real-time RT-PCR revealed that this G2/M arrest was associated with changes in mRNA expression levels of cyclin A and B. Cyclin-dependent kinase inhibitors p21^*cip*1^ and p27^*kip*1^ and the tumor suppressor p53 were only enhanced in HIE cells necessary for arresting cells in the G2/M phase and induction of differentiation. In HT-29 and Caco-2 cells, a loss of p53 expression failed to induce G2/M associated induction of differentiation. The HIE cell specific differentiation induced by BMO was a result of influencing the phosphorylation states of EGFR (epidermal growth factor receptor) and MAP kinase, i.e., ERK1/2 (extracellular signal-regulated kinase 1/2), p38-α, and Akt2 phosphorylation. These results suggest that BMO inhibited intestinal cell proliferation and altered cell cycle dynamics by affecting corresponding regulator genes and mitogen-activated protein kinase signaling. As the development and maturation of digestive and absorptive processes depends on gut differentiation processes, our *in vitro* experiments show that breed-specific BMO are natural substances influencing various parameter which may be important *in vivo* in gastrointestinal development. This, however, needs to be proven in future studies.

## Introduction

In the last decade, there has been a tremendous research interest in milk oligosaccharides, driven by the advances in chemical sciences, food technology as well as in chemical-enzymatic synthesis to produce single human milk oligosaccharides (HMO) (1, 2). This interest is primarily based upon the progress in HMO and the increasing number of studies investigating their biological functions (3–7). Currently, two HMO, e.g., 2′FL (Fucosyllactose) alone or in combination with LNnT (Lacto-N-neo-tetraose), which have been industrially produced and which are identical to their human milk counterparts, are already added to infant formula (8, 9). Oligosaccharides in general, whether derived from animals, plants or of synthetic origin, are considered to have an impact on human health, mainly through their prebiotic effects in the gastrointestinal tract. Meanwhile a whole variety of various prebiotics are added to infant formula to imitate their functions (e.g., galacto- and fructooligosaccharides (GOS and FOS), acidic plant derived carbohydrates) (10). It is often concluded that the addition of, for example, GOS and FOS to infant formula would bring them closer to human milk (10, 11). However, these components are not present in human milk (12, 13). There is no structural similarity between such prebiotic GOS/FOS and HMO. So far, no study has been carried out proving that the effects of GOS/FOS on, for example, the immune system are comparable to those of HMO. Due to this discrepancy in structure between both classes of carbohydrates, prebiotic oligosaccharides and HMO, there is currently a great interest in finding alternatives for HMO.

To receive milk oligosaccharides on a large scale which are identical to those in human milk, several strategies are currently applied. Besides chemical-enzymatic synthesis or fermentation strategies, membrane filtration is also used to separate milk oligosaccharide fractions or even individual components to investigate their potential functions in applied science (1, 2, 14, 15). Although the number and the total amount of oligosaccharides in animal milk compared to human milk is rather very low (16) it might be an interesting source to separate a few components due to the huge amount of milk available from cows, goats, or other species. In this context, an interesting aspect to investigate is whether reported differences in milk oligosaccharide compositions between various breeds (17–19) have an influence on functional processes. Hence, various questions, which currently are of great interest from a scientific and a commercial point of view, have to be addressed: Are milk oligosaccharides from various animals promising components to improve the overall health of the recipients? Do single milk oligosaccharides from animals affect the microbial composition and/or activities more efficiently than a mixture of various components and how can health effects be investigated in humans? Which specific oligosaccharides have a direct impact on intestinal or tissue target cells, i.e., on cell maturation, cell surface glycosylation or brain functions?

As we have previously reported on the effects of HMO on proliferation, differentiation and cell signal events (20, 21), the major aim of our current study was to investigate the functional effects of the milk carbohydrate fractions from various cattle breeds using different intestinal cell lines.

## Results and Discussion

### Effects of Bovine Oligosaccharides on Proliferation and Differentiation of Intestinal Cells

**Figure 1:**
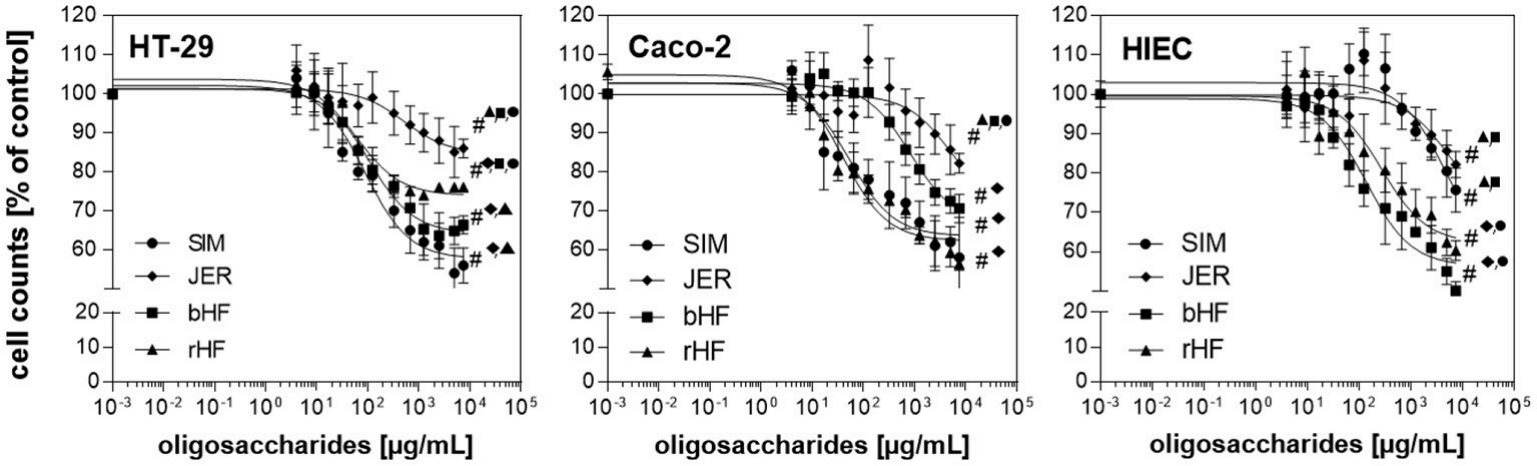
Effect of BMO on the proliferation of intestinal epithelial cells. Dose dependent inhibition effects of BMO from SIM (•), JER (♦), bHF (■), and rHF (▴) on the proliferation of HT-29, Caco-2, and HIE cells. HT-29, Caco-2 (1,500 per well) and HIE (2,500 per well) cells were incubated for 24 h. The cells were then left untreated or treated with BMO at concentrations of 0–10 mg/mL for 72 h. Results were expressed as % of controls (untreated); each value represents the mean with standard deviation (*n* = 3). ^#^ indicates significant interbreed variation at 10 mg/mL.

To address questions related to gut maturation events associated with tissue morphogenetic and cell dynamic changes, we used HT-29 and HIE cells which are intestinal cells with a lower differentiation phenotype, and Caco-2 cells which display characteristics of differentiated epithelial cells (22–25). Regarding proliferation, oligosaccharides from the breeds rHF, bHF, SIM, and JER exerted a pronounced effect in all three cell lines with highly significant interactions (*P* < 0.001) in HT-29, Caco-2, and HIE cells, respectively (Figure 1).

The growth inhibition was dose-dependent, albeit with a different magnitude in the three cell lines. Oligosaccharides from JER induced the lowest cell response in all three cell lines which was 17.6 ± 8.14% in HT-29, 16.3 ± 5.78% at the highest concentration (10 mg/mL) in Caco-2 and 17.1 ± 4.77% in HIEC. SIM-derived-oligosaccharides inhibited cell proliferation by 43.2 ± 4.9% (HT-29), 40.9 ± 5.3% (Caco-2), and 25.8 ± 5.6% (HIEC), respectively. Comparing the growth inhibition effect of BMO for the different cell types, HT-29 and Caco-2 cells appeared more sensitive to BMO than HIE cells (Figure 1).

Growth inhibition was associated with arresting cells in different cell cycle stages. Flow cytometry analysis showed that, independently of the breed, BMO were able to arrest all intestinal cell lines in the G2/M phase (Table 1).

**Table 1:** Distribution of cell cycle phases after BMO incubation.

		**HT-29**			
	**Control**	***rHF***	***bHF***	***SIM***	***JER***
sub G0/G1	0.4%	0.5%	0.3%	0.5%	0.4%
G0/G1	71.3%	49.3%^*^	53.2%^*^	56.2%^*^	48.3%^*^
S	10.9%	10.3%	10.6%	11.3%	10.4%
G2/M	14.5%	35.5%^**^	32.4%^*^	30.3%^*^	34.3%^**^
		**Caco-2**			
sub G0/G1	0.7%	0.5%	0.6%	0.6%	0.4%
G0/G1	74.9%	49.5%^*^	66.4%^*^	65.5%^*^	54.4%^*^
S	5.4%	9.2%	11.5%	12.2%	10.6%
G2/M	12.2%	39.3%^**^	34.4%^*^	31.1%^*^	37.5%^**^
		**HIEC**			
sub G0/G1	1.5%	0.8%	1.6%	1.8%	0.7%
G0/G1	69.3%	48.8%^*^	50.9%^*^	52.2%^*^	47.1%^*^
S	5.8%	9.8%	6.7%	10.2%	5.4%
G2/M	20.2%	33.8%^**^	35.4%^*^	31.1%^*^	39.3%^**^

Data are given as % of gated cells in G0/G1-, S-, and G2/M-phases. Cells were left untreated (control) or treated with 10 mg/mL of BMO for 72 h. Growth arrest was determined by flow cytometry with the DNA-staining 7-AAD. Results were expressed as means of % gated cells (n = 3). Significant differences to control cells were accepted at

* P < 0.05 and

** *P < 0.01*.

Cell cycle analysis of controls without exposure to BMO revealed that 71.3, 74.9, and 69.3% of HT-29, Caco-2 and HIE cell population was in the G0/G1 phase and 14.5, 12.2, and 20.2% in the G2/M phase. Incubation with BMO led to a reduced cell population in the G0/G1-phase and a higher cell population in G2M-phase compared to controls. However, in all cases the diminished G0/G1- and enhanced G2/M-phase were associated without significant interbreed variations.

**Figure 2:**
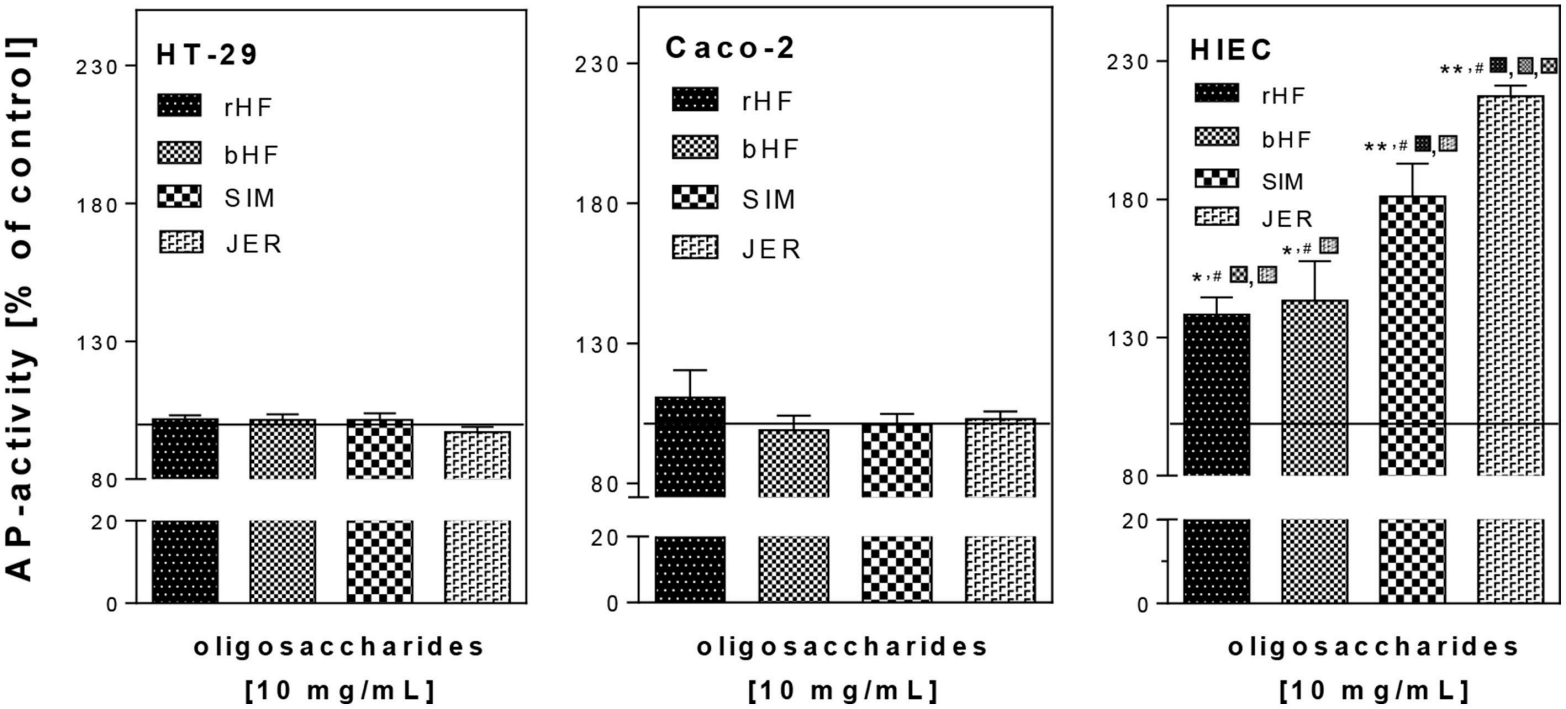
Effect of BMO on the differentiation of intestinal epithelial cells. After incubation with 10 mg/mL BMO, differentiation was determined by measuring alkaline phosphatase (AP) activity after 72 h. Results were expressed as % of control (untreated, solid line); each value represents the mean with their standard error (*n* = 3). Significant differences compared to the untreated control are indicated with *P < 0.05 and **P < 0.01; # indicates significant interbreed variation at 10 mg/mL.

As there were no detectable sub-G1 population or caspase-3-activation as markers of apoptosis (data not shown) we investigated the effect of BMO on differentiation (Figure 2). We found that treatment with 10 mg/mL BMO derived from rHF, bHF, SIM, and JER enhanced cell differentiation only in HIE cells, but not in HT-29 and Caco-2 cells. Although rHF, bHF, and SIM-derived BMO were able to increase AP activity to 129.3 ± 6.2, 132.6 ± 8.1, and 173.6 ± 5.9%, respectively, the strongest effect was found using BMO from JER with an induction of differentiation up to 217.9 ± 3.4% compared to control cells. Interestingly, the interbreed variations which were observed at inhibiting HIE cell proliferation was also found at the induction of HIE differentiation for JER- and SIM-derived oligosaccharides.

Taken together, we demonstrated that BMO induced a concentration-dependent growth inhibition in HT-29, Caco-2, and HIE cells by leading to cell arrest in the G2/M phase. However, the effects varied not only between the cell lines but also between oligosaccharides from the four different cattle breeds. HT-29 and Caco-2 cells seemed to be more sensitive to growth inhibition than HIE cells.

Previously, we obtained similar results for growth inhibition and G2/M arrest with HMO as well as with some single oligosaccharides present in both, human and bovine milk (21). Regarding the different effects on the three cell lines, one can speculate that HIE cells are more susceptible to an induction of differentiation than Caco-2 and HT29 cells. In the case of Caco-2 cells, the failure to enhance differentiation can be expected since these cells already represent a more differentiated phenotype reflected by higher basal AP activity (0.609 ± 0.013 ΔE /h/10^6^ cell) compared to HT-29 or HIE cells (0.193 ± 0.023 and 0.185 ± 0.005 ΔE /h/10^6^ cell, respectively). A phenotype-associated difference in basal AP activity is well-known (26) and supports our hypothesis.

Recently, Holscher et al. (27) confirmed our previous results (20, 22) using slightly different single oligosaccharides at the same concentrations for single HMO (1 mg/mL). Both studies show, for example, that single HMO induce differentiation even in less-differentiated cells. Only in the case of 2′FL there is a difference; here, a reason might be that Holscher et al. investigated the effects of 0.2 and 2 mg/L. In addition, in our studies we used neutral and acidic milk fractions from individual donors whereas Holscher et al. applied pooled human milk obtained from previous studies. Hence, an effect, due to Lewis blood group and secretor specific milk samples on proliferation, differentiation or apoptosis might get lost.

In contrast to our previous results using HMO (20), which induced differentiation in HT-29 and HIE cell, BMO induced differentiation only in HIE cells. The reason for this difference is not yet known, but may be due to the differences in quantity and quality of oligosaccharides present. There is a much higher number of oligosaccharides in human than in bovine milk. HMO contain primarily type 1 components (galactose linked ß1-3 to the subterminal GlcNAc, e.g., in LNT) whereas in BMO primarily type 2 structures (galactose linked ß1-4 to the subterminal GlcNAc, e.g., in LNnT) are present (28). The few oligosaccharides in bovine milk are mostly sialylated (>70%) whereas in human milk acidic components reveal only about 30 % of total oligosaccharides. Another factor responsible for the different effects of HMO and BMO could be that in human milk only N-acetylneuraminic acid-containing oligosaccharides are present whereas bovine milk contains N-acetylneuraminic acid- as well as N-glycolylneuraminic acid-bearing structures (3, 14, 17, 29).

### Effects of Bovine Oligosaccharides on Expression of Cell Cycle Regulator Genes

**Figure 3:**
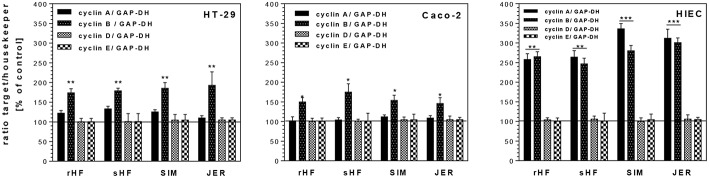
Changes of mRNA expression levels of cell cycle genes (cyclin A, B, D, E) in intestinal HT-29, Caco-2 and HIE cells using GAPDH as housekeeper gene. Cells were treated with 10 mg/mL BMO from rHF, bHF, SIM and JER after reaching a confluency of 30 % over 72 h. Then, mRNA expression levels were determined using the target gene/housekeeping gene ratio by setting the control to 100%. Values are means of the percentage of controls with their standard error (*n* = 3). Mean values were significantly different from those of the control group: *P ≤ 0.05, **P ≤ 0.01, ***P<0.001.

As shown in Figure 3 the expression level of cyclin A, a regulator for S/G2-transition, remained unchanged in HT-29 and Caco-2 cells after treatment with BMO, whereas in HIE cells cyclin A expression was stimulated significantly by all BMO. Cyclin B which is responsible for the regulation of the G2/M cell cycle transition, was markedly upregulated in all cell lines after incubation with BMO compared to controls (set to 100%). Cyclin D and E which regulate the entry of cells into and the progression through the G1 phase of the cell cycle remained unchanged in all cell lines after BMO treatment.

**Figure 4:**
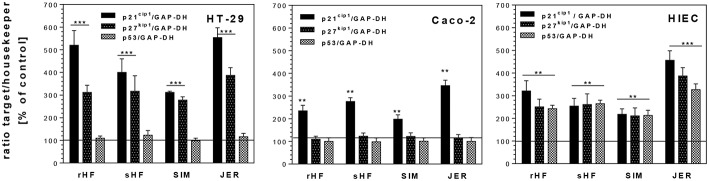
Changes of mRNA expression levels of cell cycle genes p21^*cip*1^, p27^*kip*1^, and p53 in intestinal HT-29, Caco-2 and HIE cells using GAPDH as housekeeper gene. Cells were treated with 10 mg/mL BMO from rHF, bHF, SIM, and JER after reaching a confluence of 30% over 72 h. Values are means of the percentage of controls with their standard errors (*n* = 3). Mean values were significantly different from those of the control group: *P ≤ 0.05, **P ≤ 0.01, and ***P ≤ 0.001.

In addition, we investigated whether the inhibition of cell cycle progression was accompanied by increased levels of CDKI such as p21^*cip*1^ and p27^*kip*1^ and the tumor suppressor gene p53. Both, CDKI and tumor suppressor genes, are able to induce cell cycle arrest in the G1 or G2 phase and/or induce differentiation. Treatment of the undifferentiated cell lines HT-29 and HIE with BMO resulted in an enhanced expression of p21^*cip*1^ and p27^*kip*1^ (Figure 4). In HT-29 cells, 10 mg/mL of rHF, bHF, SIM and JER-derived oligosaccharides enhanced expression of p21^*cip*1^ 5.3-, 4.3-, 3.2-, and 5.6-fold, respectively, and that of p27^*kip*1^ 3.0-, 3.1-, 2.8-, and 4.1-fold, respectively. In HIE cells, rHF-, bHF-, SIM-, and JER-derived oligosaccharides enhanced expression of p21^*cip*1^ 3.3-, 2.5-, 2.3-, and 4.5-fold, and of p27^*kip*1^ 2.4-, 2.5-, 2.3-, and 4.0-fold, respectively. In contrast to HT-29 and HIE cells, Caco-2 cells responded only with an increased p21^*cip*1^ mRNA level but p27^*kip*1^ levels remained unchanged after BMO exposure.

The observed G2/M arrest in the cells was also based on an increased expression of the CDK inhibitors and of p21^*Cip*1^ and p27^kip1^ expression. Furthermore, p53, a transcriptional regulator of several cell cycle regulating genes, is able to regulate G1 or G2 transition (30). Interestingly, enhanced p53 mRNA levels were found only in HIE cells after incubation with 10 mg/mL of rHF-, bHF-, SIM-, and JER-derived oligosaccharides.

The effects of BMO observed on proteins responsible for cell cycle progression seemed to be regulated in a similar way as observed with HMO for HT-29 and Caco-2 cells (21). Similar to HMO, BMO from the different cattle breeds induced a p53-independent p21^*cip*1^ expression with changes of cyclin B. These changes in expression levels were associated with growth inhibition and G2/M arrest. However, our data suggest that BMO-mediated up-regulation of cyclins and CDKIs involves a p53-independent pathway, as HT-29 cells lack functional p53. However, in contrast to our previous observations using HMO, these effects were not associated with an increase in AP activity when HT-29 cells were exposed to BMO (21). Similar to the results obtained by growth-inhibition curves (see above), qualitative and quantitative differences in oligosaccharide pattern between species and even between breeds might be the reason for divergent cell response.

### Influence of Signal Transduction Pathways

**Figure 5:**
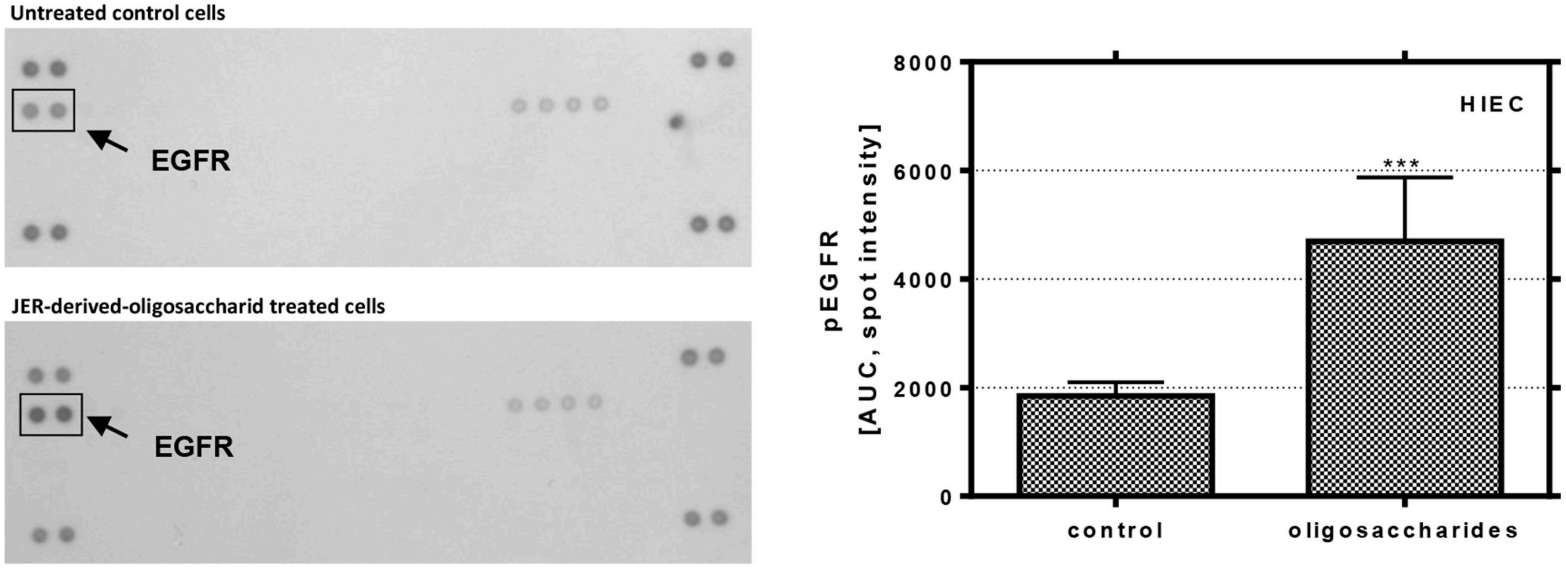
Phosphorylation of EGFR in HIE cells. Cells were treated with 10 mg/mL JER-derived-oligosaccharides for 10 min. Lysate was prepared according to the manufacturer's instructions. Phospho-receptor tyrosine kinase array was used to detect phosphorylation of these receptor tyrosine kinases in HIE cells. The signal was detected by chemiluminescence and the spot intensity is shown. Values are means of the percentage of controls with their standard errors (*n* = 2). Mean values were significantly different from those of the control group (***P ≤ 0.001) [AUC, area under the curve; EGFR, epidermal growth factor receptor].

In order to analyze the effects of BMO on the activation of signal transduction pathways in more detail, the phosphorylation of different growth factor receptors and molecular targets was investigated for HIE cells, representing phenotypical undifferentiated cells, in which BMO were able to induce differentiation, a key event in gut maturation (Figure 5). The observed effects on proliferation and differentiation with associated changes in expression levels of cyclins, CDKI and p53 are a consequence of activation or inactivation of different signal cascades. Therefore, we further investigated the influence of the most effective BMO in differentiation (JER-derived oligosaccharides) on the phosphorylation state of several receptors and MAP kinases in HIE cells using protein profiling arrays to detect different phosphorylation events. JER-derived oligosaccharides (10 mg/mL) induced a phosphorylation of the ERFR by up to 265 ± 7% compared to untreated control cells (100%) indicating that BMO could interact with the growth factor receptor. This effect was EGFR specific because no other receptor phosphorylation was observed Figure 5).

**Figure 6:**
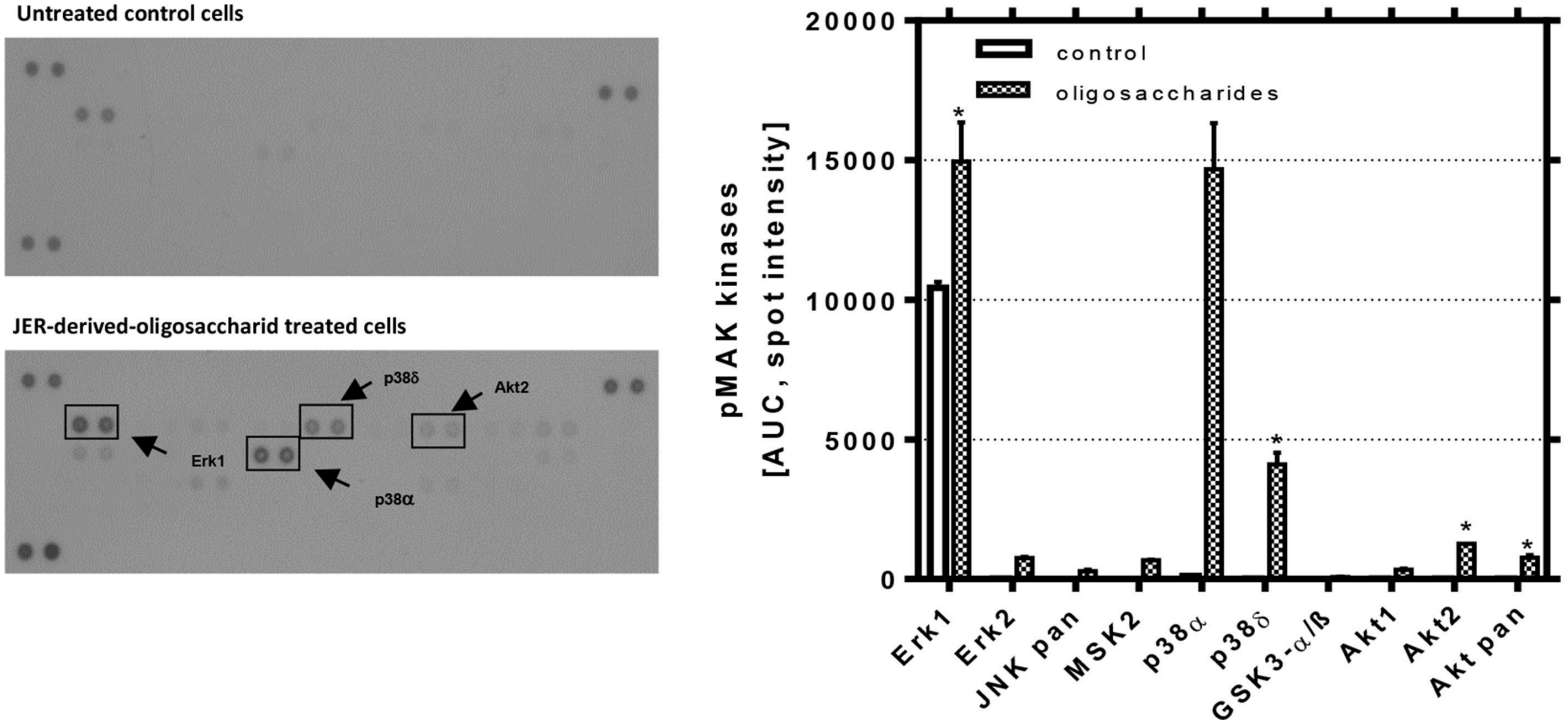
Detection of phosphorylated mitogen-activated protein kinases in HIE cells. HIE cells were treated with JER-derived-oligosaccharides for 30 min. After incubation, 300 μg cell lysate were used for each assay. Array signals from scanned X-ray film images (left: representative blot) were analyzed using image analysis software and expressed as spot pixel density. Values are means with their standard errors (*n* = 2). Mean values were significantly different: *P ≤ 0.05 and ***P < 0.001 [AUC, area under the curve; pMAP, phosphorylated MAPK].

As a consequence of receptor phosphorylation different signal pathways could be induced. Hence, we used a MAPK array to investigate how and to which extent JER-derived oligosaccharides are able to induce downstream events from EGFR signaling. As shown in Figure 6, the analysis of these signaling pathways revealed that p38 MAPK, extracellular signal-regulated kinase (ERK) 1 and 2 and protein kinase B (Akt) play a role. Phosphorylation and activation of different p38 MAPK subtypes, especially p38α and p38δ in HIE cells, were induced by JER-derived oligosaccharides (10 mg/mL). In addition, we showed that both PKB and ERK were phosphorylated when cells were treated with oligosaccharides from JER. PKB-β/Akt (Akt2) and Akt pan, two growth factor-regulated protein kinases, and the downstream kinase ERK1 have emerged as critical enzymes in signal transduction pathways involved in cell proliferation and differentiation (31).

Recent studies suggest that the EGFR pathway is not simply a growth promoting signaling pathway, but phosphorylated EGFR (pEGFR) also mediates p21^*cip*1^ expression and growth arrest or apoptosis (32). From our study two major findings have emerged similar to that found previously with HMO (21): we observed (i) that JER-derived BMO were able to significantly induce EGFR phosphorylation and (ii) we confirmed that the EGFR/Ras/Raf/ERK pathway was involved. Based upon these observations we conclude that BMO-caused differentiation is a consequence of p53-dependent p21^*cip*1^ expression and stabilization via EGFR and p38 kinase phosphorylation.

**Figure 7:**
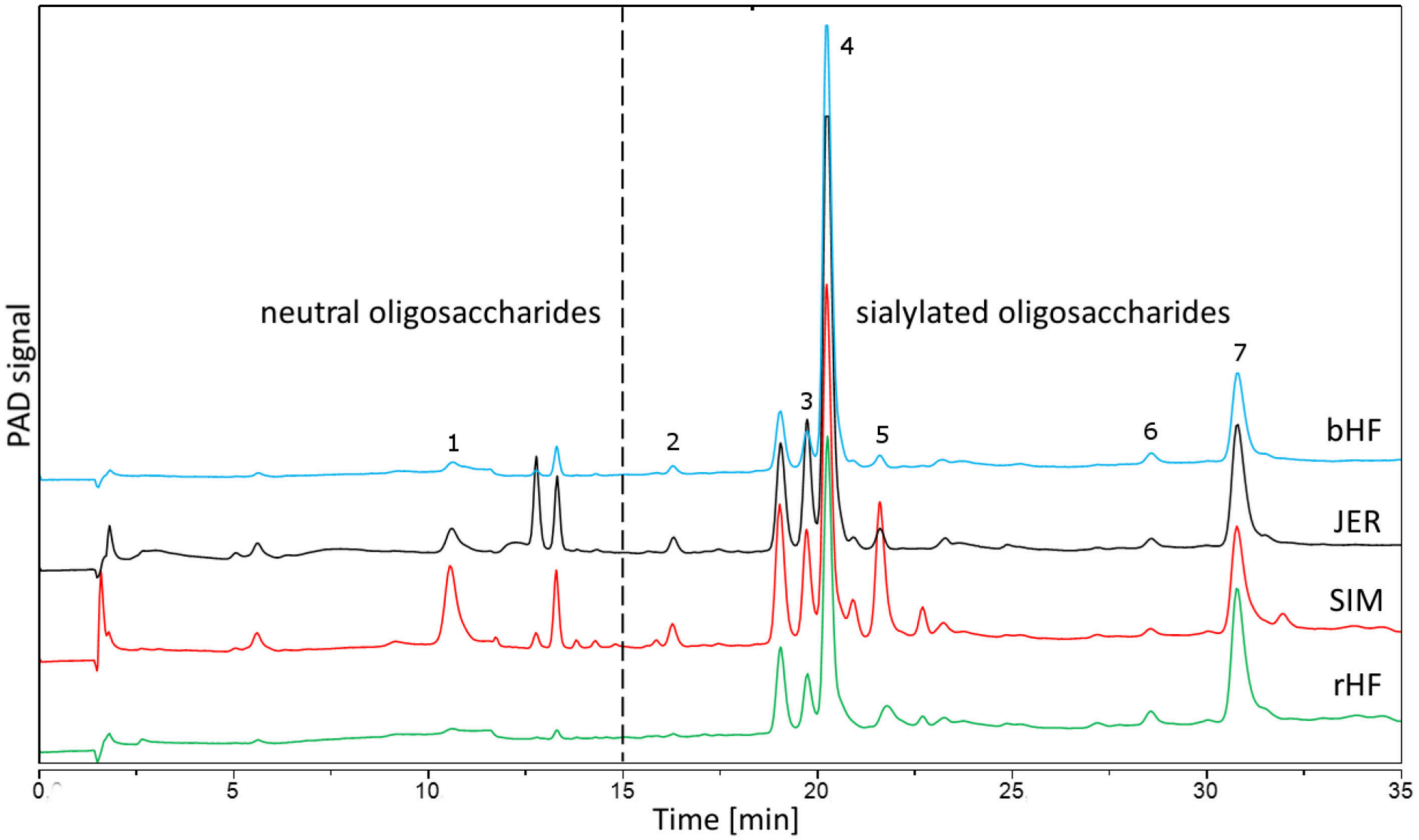
Chromatographic separation using HPAEC-PAD of BMO from four different cattle breeds. BMO from early bovine milk (colostrum) were isolated as described in the Method section. Identified peaks are 1: galactosyl-lactose; 2: *N*-acetylneuraminic acid; 3: 6′-N-acetylneuraminyl-lactose; 4: 3′-*N*-Acetylneuraminyl-lactose; 5: 6′-*N*-acetylneuraminyl-*N*-acetyllactosamin; 6: *N*-glycolyl-neuraminyl-lactose; 7: disialyl-lactose. bHF, German Holstein cattle (Black Pied); rHF, German Holstein cattle (Red Pied); SIM, German Simmental; JER, Jersey.

In conclusion, we identified that BMO from different cattle breeds were able to induce growth arrest and differentiation of non-transformed HIE cells by modulating EGFR signal pathways, and cell cycle associated gene expression in a similar way as was shown for HMO (20, 21). Whether differences with regard to the magnitude of effects dependent on breed specific BMO (as shown in Figure 7) has implications for the intestinal growth regulation in infants is not yet known and requires further investigation. Overall, differences in composition and diversity of milk oligosaccharides will most likely have functional consequences. To proof whether these data translate into the human infant situation, rigorous preclinical and clinical trials would be required to come to a clear conclusion.

## Materials and Methods

### Preparation of Oligosaccharides From Bovine Milk

Milk samples were collected during regular milking from dairy cattle breeds (for milk production) in Germany at the Research Station “Oberer Hardthof” at the Justus-Liebig University Giessen, kindly provided by Prof. G. Erhardt (Institute for Animal Breeding and Genetics). According to the German Animal Welfare Law (released on 05/18/2006) no notification or approval by the Animal Protection Unit of the Regional Council of Gießen (Germany) was necessary for this study. Thus, we included four breeds, i.e., German Holstein cattle (Black Pied, bHF and Red Pied, rHF), German Simmental (SIM) and Jersey (JER). Cows were milked within the first 48 h of lactation and samples were frozen at −20°C until analysis. BMO were isolated as described previously (20, 21). Briefly, after centrifugation, the lipid layer was removed, and proteins were precipitated from the aqueous phase using ice-cold ethanol. Lactose was removed by gel filtration on Sephadex G-25 (Pharmacia Biotech, Uppsala, Sweden). Pooled oligosaccharide fractions were freeze-dried, their composition was analyzed by high pH anion-exchange chromatography with pulsed amperometric detection (HPAEC-PAD) using the conditions described previously (33). For cell culture studies bovine oligosaccharides from the different breeds (Figure 7) were used at concentrations up to 10 mg/ml in the corresponding culture media with 10% fetal calf serum (FCS), 2 mM glutamine, 100 units/mL penicillin and 100 mg/mL streptomycin (Invitrogen, Karlsruhe, Germany).

### Cell Culture

The human colon cancer cell line HT-29 and Caco-2 was obtained from the American Type Culture Collection (ATCC, Rockville, MD, USA). The fetal intestinal colon cell line HIEC was generously donated by J.F. Beaulieu (Department of Anatomy and Cell Biology, Faculty of Medicine, Université de Sherbrooke, Sherbrooke, Quebec, Canada). HT-29, Caco-2 and HIE cells were used between passages 45–50, 40–50, and 10–15, respectively. Cells were cultured in 75 cm^2^ tissue culture flasks (Renner, Darmstadt, Germany) in RPMI 1640 (HT-29 and HIEC) or DMEM (Caco-2) supplemented with 10% fetal calf serum (FCS), 2 mM glutamine (Invitrogen, Karlsruhe, Germany), 100 units/mL penicillin and 100 mg/mL streptomycin (Invitrogen, Karlsruhe, Germany). The cultures were kept in a humidified atmosphere of 5% CO_2_ at 37°C. Cells were passaged at preconfluent densities using 0.05% trypsin and 0.5 mM EDTA (Invitrogen, Karlsruhe, Germany).

### Measurement of Cell Dynamics

Proliferation of cells was determined after 72 h incubation of adherent cells using the 3-(4,5-dimethylthiazol-2-yl)-2,5-diphenyl-tetrazoliumbromide (MTT)-assay as has been described previously (20). Cell numbers were determined based on a calibration curve using cell counts between 500 and 30,000. Differentiation was determined by alkaline phosphatase activity on 25 cm^2^-culture flasks (Renner, Dannstadt, Germany). After having reached 30–40% confluency, cells were incubated for 72 h in the presence or absence (control) of oligosaccharides (pH 7.4) as has been previously described (20). Alkaline phosphatase activity was measured as ΔE/h/10^6^ cells and the controls were set to 100%. Cell Cycle Analysis of intestinal cells (HT-29, Caco-2, and HIEC) were measured by flow cytometry. Therefore, cells were seeded at a density of 50,000 onto 6-well culture flasks and allowed to adhere for 24 h. Thereafter, the medium was replaced and incubated for another 24 h in the presence or absence of oligosaccharides. Cell cycle analysis was performed using a FACScan (Becton Dickinson, San Jose, CA, U.S.A.) and the software BD CellQuest™ Pro (Version 1.41.) for data analysis (20, 21).

### Measurement of Gene Expression and Signaling Pathways

Total RNA isolation from intestinal cells, cDNA synthesis and real-time PCR were performed as described earlier (21). Messenger RNA expression of cell cycle genes such as cyclins (cyclin A, B, D, and E), CDKI and p53 were determined in relation to the expression of the housekeeping gene GAPDH; results from untreated cells were set at 100% (21). Receptor phosphorylation studies were made with preconfluent (70–80%) HIE cells. Cells were incubated in DMEM with 10% FCS for 24 h. Subsequently, JER-derived oligosaccharides were added in concentrations indicated in the legend of respective figures. The Proteome Profiler™ array—human phospho-RTK array kit to identify the phosphorylation of 42 different RTKs was used according to the manufacturer's instructions (R&D Systems, Minneapolis, MN, USA) as has been done previously (21). MAP-Kinase phosphorylation studies were made with preconfluent (70–80%) HIE cells. After incubation of intestinal cells with JER-derived oligosaccharides (10 mg/mL), 300 μg of total protein were used for the Human Phospho-MAP array®(R&D Systems; Heidelberg, Germany) according to the manufacturer's protocol (21).

### Statistical Analysis

For each variable at least three independent experiments were carried out and the results were expressed as mean values with their standard errors (mean ± SEM) or standard deviation (mean ± SD). Statistical differences were tested by one-way ANOVA with Bonferroni's *post hoc* test and differences were considered significant at ^*^P < 0.05, ^**^P < 0.01, and ^***^P < 0.001 to controls or indicate significant interbreed variation at 10 mg/mL with bHF (#a), rHF (#b), SIM (#c), and JER (#d). Two-way ANOVA was used to test significant interactions between concentrations and breeds (*P* < 0.001) in HT-29, Caco-2, and HIE cells, respectively. All analyses were carried out with the GraphPad Software Prism 6.02 (San Diego, CA, USA).

## Author Contributions

CK, SR, and SK designed the study. SK performed the laboratory work and statistical analysis. SK and SR discussed the interpretation of the data. SK wrote the draft version of the manuscript. SR, CK, and SK had primary responsibility for the final content.

### Conflict of Interest Statement

The authors declare that the research was conducted in the absence of any commercial or financial relationships that could be construed as a potential conflict of interest.

## Abbreviations

BMO: bovine milk oligosaccharides
HMO: human milk oligosaccharides
2'FL: 2'Fucosyllactose
GAPDH: Glycerinaldehyd-3-phosphat-Dehydrogenase
GlcNAc: N-acetylglucosamine
LNT: Lacto-N-tetraose
LNnT: Lacto-N-neo-tetraose
GOS: galactooligosaccharides
FOS: fructooligosaccharides
HPAEC-PAD: High Performance Anion Exchange Chromatography with Pulsed Amperometric Detection
NeuAc: N-acetylneuraminic acid
HexNAc: *N*-acetylhexosamine
bHF: German Holstein cattle (Black Pied)
rHF: German Holstein cattle (Red Pied)
SIM: German Simmental
JER: Jersey
CaCo-2 cells: human colonic carcinoma cells
HIEC: human intestinal epithelial cells
HT-29 cells: human intestinal tumor cells
AP: alkaline phosphatase
CDKI: cyclin-dependent kinase inhibitors
EGFR: epidermal growth factor receptor
MTT: 3-(4, 5-dimethylthiazol-2-yl)-2,5-diphenyltetrazoliumbromide
AUC: area under the curve
pMAP: phosphorylated Mitogen Activated Protein Kinases.
